# Evaluating the Effects of Cryopreservation on the Viability and Gene Expression of Porcine-Ear-Skin Fibroblasts

**DOI:** 10.3390/genes14030751

**Published:** 2023-03-20

**Authors:** Jiacheng Cao, Yingyu Xie, Jing Wang, Yongjie Huang, Xiaohan Zhang, Tianfang Xiao, Shaoming Fang

**Affiliations:** College of Animal Science (College of Bee Science), Fujian Agriculture and Forestry University, Fuzhou 350002, Chinatfxiao@fafu.edu.cn (T.X.)

**Keywords:** fibroblast cultures, cryo-stimulation, cell recovery and survival, RNA-sequencing

## Abstract

Owing to the inherent heterogeneity and plasticity of fibroblasts, they are considered as the conventional biological resources for basic and clinical medical research. Thus, it is essential to generate knowledge about the establishment of fibroblast cultures and the effects of cryopreservation processes on their biological characteristics. Since the pig (*Sus scrofa*) possesses numerous genetic, physiological, and anatomical similarities with humans, porcine fibroblasts are naturally regarded as useful analogues of human fibroblasts. Nonetheless, less attention has been given to the alterations in viability and gene expression of cryopreserved porcine fibroblasts. In this study, we aimed to obtain fibroblasts from porcine ear skin and evaluate the effects of cryopreservation on the cell survival, proliferation, and gene expression profiles of the fibroblasts by trypan-blue-staining assay, Cell Counting kit-8 (CCK-8) assay, and RNA-sequencing analysis, respectively. Our results suggested that morphologically stable fibroblast cultures can be constructed from pig-ear skin. The post-thaw survival rate of the cryopreserved fibroblasts at 0 h and 24 h was over 90%. The proliferative activity of the cryopreserved fibroblasts was similar to that of the non-cryopreserved fibroblasts after 7 days of in vitro culture, which suggested that cryopreservation did not influence the viability. The RNA-sequencing analysis indicated that this should be attributed to the 867 differentially expressed genes (DGEs) identified, which are involved in molecular process related to cell recovery and survival after cryo-stimulation. In addition, eight important DEGs *BMP2*, *GDF15*, *EREG*, *AREG*, *HBEGF*, *LIF*, *IL-6*, and *HOX-7* could potentially be applied to improve the efficiency of fibroblast cryopreservation, but comprehensive and systematic studies on understanding the underlying mechanisms responsible for their modulatory roles are urgently needed.

## 1. Introduction

Fibroblasts reside in the connective tissues of most organs, which possess naturally heterogeneity and plasticity [1]. Accumulating evidence derived from basic and clinical medical studies has demonstrated their important roles in tissue repair and fibrosis, immune surveillance and inflammation, blood-vessel function, and cancer progression [2]. Fibroblast studies are facilitated by the establishment of diverse fibroblast cell lines, and cryopreservation is recognized as an efficient technique for the long-term storage of fibroblast cell lines. Accordingly, a large number of human and animal studies have been performed to describe the establishment of fibroblast cultures and how cryopreservation affects their preservation and quality [3,4,5]. For instance, Aydoğdu et al. systematically summarized the procedures for the isolation, culture, and cryopreservation of fibroblasts derived from human skin [6]. Seluanov et al. provided a protocol for the isolation and culture of fibroblasts from rodent skin and lungs [7]. Bai et al. successfully established a pig-fibroblast-cell bank by using the approach of primary explantation and cryopreservation [8]. In addition, Naaldijk et al. reported that the cryopreservation of human fibroblasts in an adherent state significantly diminishes their subsequent growth potential owing to the fact that adherent cells are more vulnerable to the mechanical stress induced by cryopreservation [4]. Praxedes et al. indicated that compared to non-cryopreserved rodent fibroblasts, the cryopreserved fibroblasts exhibited lower mitochondrial membrane potential and viability [9].

Since the pig exhibits great genetic, physiological, and anatomical similarities with human, porcine fibroblasts are reasonably considered as potentially optimal alternatives to human fibroblasts in various studies [10,11,12]. Sood et al. suggested that the pig is a promising model for the study of human pathologic scarring as the in vitro behavior of porcine dermal fibroblasts closely mirrored that of human hypertrophic scar fibroblasts under similar conditions [13]. Walker et al. revealed that nuclear mechanosensing drove chromatin remodeling in persistently activated fibroblasts from porcine aortic valves shared similar signatures with human fibroblasts from fibrotic valves, which emphasized the potential relevance of altered mechanosensing in human-fibrosis-disease progression [14]. Smatlikova et al. generated a pig model for human Huntington’s disease (HD) and suggested that age-related oxidative changes in isolated fibroblasts enlightened the study on molecular mechanisms of HD pathophysiology [15].

With the extensive utilization of porcine fibroblasts in different studies, cryopreservation is crucial for establishing the cell bank to provide permanent and sufficient cell sources. The freezing-container method, machine-controlled cryopreservation, and vitrification are commonly used for cryopreserving fibroblasts. Previous studies investigated how different cryopreservation methods affected the cellular activities and functionalities of fibroblasts. For instance, Naaldijk et al. compared the effects of different machine-based and manual cryopreservation methods on the post-thaw survival and proliferative capacity of human fibroblasts, and suggested that the freezing-container method was the most effective solution [4]. Wang et al. revealed that mouse (*Mus musculus*) fibroblasts’ post-thaw recovery rate under slow cryopreservation was significantly higher than that under rapid freezing [16]. Costa et al. indicated that both direct vitrification in cryovials and solid-surface vitrification were effective in the preservation of agouti (*Dasyprocta leporina Linnaeus*) fibroblasts, but led to an increase in the number of perinuclear halos [17]. However, fewer studies have focused on the effects of cryopreservation on the specific biological characteristics of porcine fibroblasts. In the current study, we isolated and cultured fibroblasts from porcine ear skin. Next, the viability and gene expression of cryopreserved porcine fibroblasts were assessed via trypan-blue-staining assay, CCK-8 assay, and transcriptome sequencing analysis. Our findings could offer important insights into how cryopreservation affects porcine fibroblasts and provide valuable information for improving the outcome of fibroblast cryopreservation. 

## 2. Materials and Methods

### 2.1. Porcine-Ear-Skin-Sample Collection

Ear-peripheral-skin samples (about 1–2 cm^2^ in size) were randomly collected from 20 healthy Guanzhuang spotted piglets (female, 28 days of age) by using a sterilized scalpel and pliers after sterilization with 75% alcohol on the Guanzhuang spotted-pig farm, Longyan, China. Subsequently, the samples were placed into sterile tubes containing DMEM supplemented with ampicillin (100 U/mL) and streptomycin (100 g/mL) at 4 °C for 1 h transportation. All procedures involving animals in our study were in accordance with the guidelines for the care and use of experimental animals and approved by Animal Care and Use Committee (ACUC) of Fujian Agriculture and Forestry University (NO. PZCASFAFU2022069).

### 2.2. Cell Cultures

Tissue-explant culture and enzymatic digestion methods were used to initiate the primary culture, with some minor modifications [8]. In brief, the collected samples were washed three times using phosphate buffered saline (PBS) and chopped into small 1 mm^3^ explants, which were subsequently seeded into cell-culture flasks containing Dulbecco’s Modified Eagle’s Medium (DMEM) supplemented with 10% fetal bovine serum at 37 °C under a humidified environment with 5% CO_2_ and 95% air. The medium was exchanged every other day before subculture. When the cell confluence reached 80–90%, they were passaged with 0.25% trypsin and separated into prepared culture flasks at a ratio of 1:3 for further analysis.

### 2.3. Microbial Contamination Detection

Daily assessment was performed under light microscopy to identify the bacterial and fungal contamination in the subcultured cells incubated with DMEM containing 10% FBS free of antibiotics–antimycotics. A nested polymerase chain reaction (PCR) kit (PCR Mycoplasma Detection Set, TaKaRa, Japan) was used to detect the presence of mycoplasma.

### 2.4. Identification of Fibroblasts

Cells from the third passage (P3) derived from 15 individuals were used for the identification of fibroblasts by morphological observation and immunofluorescence analyses [18]. The morphological characteristics of the cells were detected under an inverted light microscope (TS100, Nikon, Japan) to observe cellular and nuclear shapes and cytoplasmic extensions. For further morphological confirmation, the cells were subjected to vimentin-immunofluorescence-staining assay. The cells were fixed in 4% paraformaldehyde for 20 min at room temperature, and then washed with cooled PBS for three times. Subsequently, the cells were resuspended in antigen-retrieval buffer (100 mM Tris, 5% urea, pH 9.5) and incubated for 24 h, and then washed three times using PBS and permeabilized in 0.4% Triton X-100 for 1.5 h. To block the non-specific binding of the antibodies, the cells were treated with a solution containing 5% BSA, PBS, and 0.1% Tween 20 for 1.5 h. Subsequently, the cells were incubated with rabbit anti-vimentin antibody (1:200, AF7013, Affinity Biosciences, Cincinnati, OH, USA) at 4 °C for 24 h, followed by incubation with Alexa Fluor^®^ 488-coupled goat anti-rabbit IgG (1:250, S0018, Affinity Biosciences, USA) in a dark room for 1 h. Nuclei were counter-stained with 1.0 mL DAPI for 3 min and observed under a fluorescence microscope (TE2000, Nikon, Japan).

### 2.5. Cryopreservation, Recovery, and Cell-Viability Measurement

The P3 cells were subjected to slow freezing in DMEM supplemented with 10% DMSO 10% FBS, and 0.2 M sucrose to obtain a final concentration of 3.0 × 10^6^ cells/mL. Subsequently, 1 mL of the cell suspension was divided into each cryotube labeled with ID, passage number, and date, and kept at 4 °C for 30 min to equilibrate the DMSO. Subsequently, the cells were transferred to the freezing-container Mr. Frosty system^®^ (Thermo Scientific, Waltham, MA, USA) for 12 h and, later, to a freezer at −80 °C while maintaining a cooling rate of 1 °C/min. After reaching −70 °C, they were quickly transferred to liquid nitrogen for long-term storage. After 10 days of cryopreservation, the cells were thawed by incubation at 25 °C for 1 min and at 37 °C for 4 min. Next, DMEM containing 0.2 M sucrose was added into the tubes and kept at 4 °C for 15 min. After centrifugation at 600× *g* for 10 min to remove the DMSO, the thawing cells were revived in the fresh culture medium for subsequent analysis.

To evaluate the effect of cryopreservation on viability of fibroblasts, the post-thawing P3 cells derived from three randomly selected individuals were subjected to survival-rate measurement by using trypan-blue-staining assay. Subsequently, relative concentration of viable cells of P3 cells before freezing and post-thawing were assessed according to the instructions of the commercial cell counting kit-8 (Merck, Darmstadt, Germany). After 7 days of culture, the growth curve was generated by using the culture time and OD value at 450 nm.

### 2.6. RNA-Sequencing Analysis

To uncover the alterations in the gene-expression profiles triggered by cryopreservation, RNA-sequencing analysis was performed by using the aforementioned cryopreserved and non-cryopreserved cells at the end of 7 days of culture. The total RNA was extracted using ZR RNA MiniPrep kit (Zymo research, Irvine, CA, USA) according to the manufacturer’s instructions. The RNA integrity and concentration were determined by 1% agrose gel and NanoDrop 2000 Spectrophotometer (Thermo Scientific, USA), respectively. A total of 2 μg of qualified RNA was used for cDNA-library construction by using the NEB Next Ultra II RNA Library Prep Kit for Illumina (NEB, Ipswich, MA, USA) according to the manufacturer’s recommendations. The constructed library was sequenced on an Illumina HiSeq 4000 platform (Illumina Inc., San Diego, CA, USA) for 2 × 150-bp reads.

Clean data were generated by removing the adapter sequences, ploy-N reads, and low-quality reads in raw data through the in-house Perl scripts. Subsequently, Q30, GC content, and sequence-duplication level of the clean data were calculated. The clean reads were aligned to the reference genome *S. scrofa* 11.1 by the HISAT2 (v2.0.4) [19]. StringTie (v1.3.1) was applied for transcriptome assembly [20]. After gene-expression level was quantified by fragments per kilobase per million mapped reads (FPKM); DESeq2 (v1.10.1) was used to identify differentially expressed genes (DEGs) between the cryopreserved and non-cryopreserved cells with the criteria of fold change ≥1.5 and FDR adjusted *p* < 0.05 [21]. The gene ontology (GO) and Kyoto Encyclopedia of Genes and Genomes (KEGG) analyses were performed by using clusterProfiler R package [22].

### 2.7. Quantitative Real-Time PCR Validation

To confirm the results of transcriptome sequencing, 8 specific DEGs related to the effects of cryopreservation on fibroblasts were chosen for verification by qRT-PCR. The primer sequences are shown in Table S1. PrimeScript RT Reagent Kit (TaKaRa, Shiga, Japan) was used to synthesize cDNA from 1.5 μg of total RNA for each sample. The qRT-PCR analysis was performed using the ABI 7500 Real-Time PCR System (ThermoFisher, Waltham, MA, USA) with Power SYBR Green PCR Master Mix (ThermoFisher, Waltham, MA, USA). The relative expression level of each gene was normalized to the endogenous control gene *GAPDH*, and expression ratios were calculated by using the 2^−ΔΔCt^ method.

### 2.8. Statistical Analysis

The normality and variance homogeneity of all data were first checked by Shapiro–Wilk test and Levene’s test, respectively, and then deposed to Student’s *t*-test in R software (v4.1.1). False-discovery rate (FDR) adjusted *p* < 0.05 was set as statistical significance threshold. Both R and GraphPad Prism 8 software were used for data visualization.

## 3. Results

### 3.1. Identification of the Fibroblasts and Cellular-Viability Measurement

**Figure 1:**
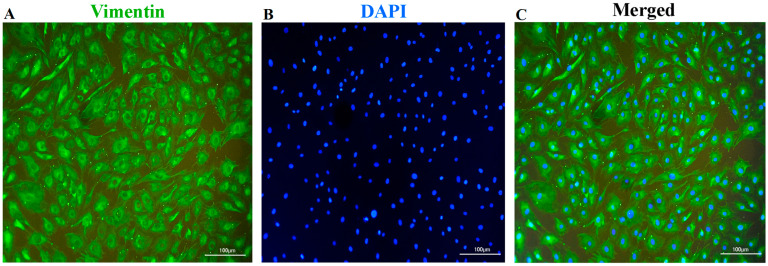
Vimentin-immunofluorescence staining of cells. (**A**). Cells stained by vimentin antibody. (**B**). Nucleus labeled with DAPI. (**C**). Merged images.

**Figure 2:**
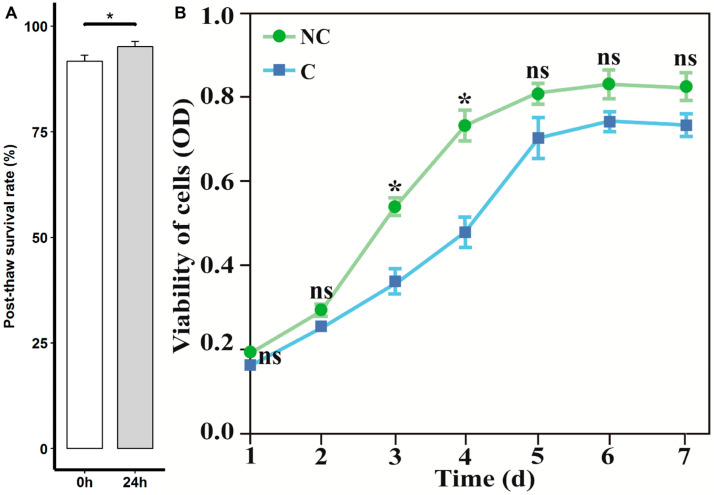
The cellular-viability measurement of fibroblasts. (**A**). The post-thaw survival rate of cryopreserved cells at 0 h and 24 h. (**B**). The growth curves of cryopreserved (C) and non-cryopreserved (NC) cells. “*” represents adjusted *p* < 0.05; “ns” indicates non-significant differences.

In the cultures, the cells presented a typical fusiform morphology and possessed oval nuclei, were preliminarily observed by light microscopy (Figure S1). To further confirm the cell type, the cells were subjected to immunofluorescence staining. As shown in Figure 1, the cells exhibited the evident features of fibroblasts, characterized by abundant vimentin in the cytoplasm, and a spindle-like-shape nucleus. Moreover, the signs of microbial contamination in the cell cultures free of antibiotics and antifungals were not detected by either light microscope observation or nested PCR. To determine the effect of cryopreservation on the cellular viability, the post-thaw survival rates of the fibroblasts were first assessed by using trypan-blue staining. The post-thaw survival rates at 0 h and 24 h were 91.72 ± 1.42% and 95.15 ± 1.26%, respectively (Figure 2A). Subsequently, the growth curves of the cryopreserved and non-cryopreserved cells were constructed by using the CCK-8 assay. From the 7-day culture of the cells, an S-shaped growth curve was observed in the two groups (Figure 2B). Except for the exponential phase (days 3 and 4), the cryopreservation did not significantly affect the cellular viability.

### 3.2. RNA-Sequencing Data and Alignment-Quality Assessment

To assess the molecular alterations of fibroblasts in response to cryopreservation, RNA sequencing was performed. As shown in Table 1, after removing the low-quality reads and adapters, a total of 294.73 million clean reads were obtained from all the samples. On average, 47.59 million and 50.64 million clean reads were obtained from the cDNA libraries of cryopreserved and non-cryopreserved cells, respectively. The alignment-quality assessment indicated that the Q30 value of each group was over 94%. The GC and AT contents of each group were almost equal. Next, clean reads were aligned with the reference genome *S. scrofa* 11.1 by HISAT2. A total of 136.95 million clean reads from the cryopreserved cells were successfully mapped. Regarding the non-cryopreserved cells, 145.74 million clean reads were successfully mapped. Among these, 132.86 million and 140.74 million clean reads were uniquely mapped, respectively. Both the mapping efficiency and the uniquely mapped efficiency of each group were higher than 90%. These results indicated that the sequencing data were high quality and reliable, and could be used for subsequent analysis.

### 3.3. Differential Expression Genes (DEGs) of Fibroblasts in Response to Cryopreservation

**Figure 3:**
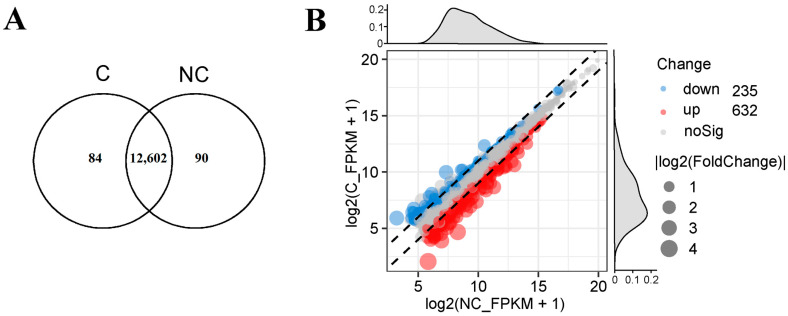
The gene-expression profiles in cryopreserved (C) and non-cryopreserved (NC) cells. (**A**). The common and specific genes in cryopreserved and non-cryopreserved cells. (**B**). The differential expressed genes (DEGs) between cryopreserved and non-cryopreserved cells.

The gene-expression profiles of the two groups of cells were composed of 12,776 genes, of which 12,602 were common genes, 84 were specific to the cryopreserved group, and 90 were uniquely found in the non-cryopreserved group (Figure 3A). Subsequently, 867 DEGs were identified with a fold change ≥1.5 and FDR-adjusted *p* < 0.05. Among these, 632 genes were up-regulated, while 235 genes were down-regulated (Figure 3B, Table S2).

### 3.4. Cryopreservation-Induced Molecular-Functional Profile Alterations of Fibroblasts

**Figure 4:**
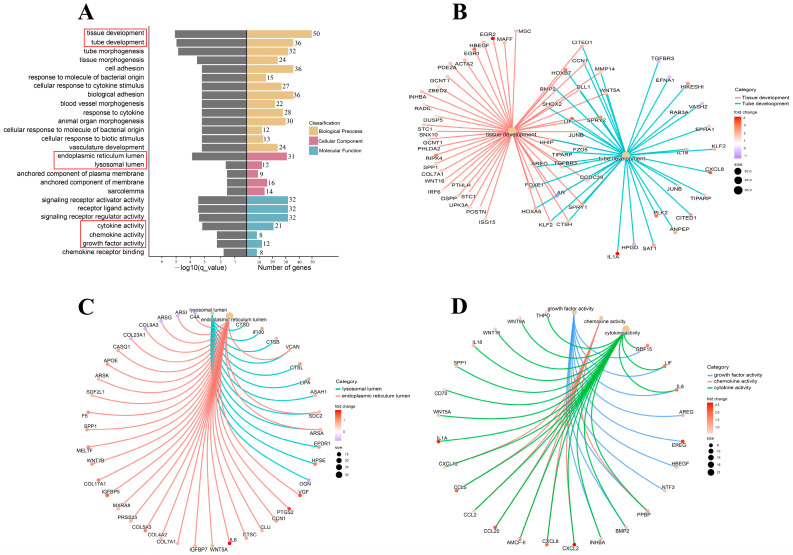
GO enrichment analysis of DEGs. (**A**). GO functional items enriched by DEGs. (**B**). DEGs enriched in tissue and tube development. The color changes of the dots correspond to fold changes of DEGs. The different color connections represent DEGs participating in different molecular processes. (**C**). DEGs enriched in endoplasmic reticulum and lysosomal lumen. (**D**). DEGs enriched in cytokine, chemokine, and growth-factor activity.

**Figure 5:**
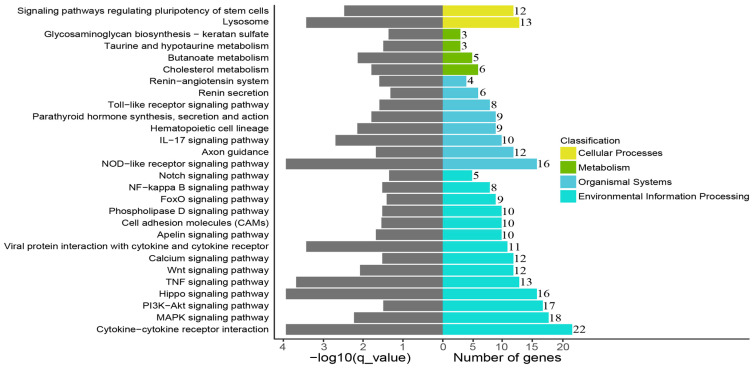
KEGG-pathway enrichment analysis of DEGs.

To explore the molecular-process alterations of the fibroblasts induced by cryopreservation, the DEGs were subjected to GO enrichment analysis. The results showed that the DEGs were enriched in biological process, cellular component, and molecular function. Each of these categories consisted of 169, 7, and 18 subcategories, respectively (Figure 4A, Table S3). In the biological process category, tissue and tube development were the two highest-ranked functional terms in terms of enrichment with several important genes, such as *BMP2*, *HOXB7*, and *LIF* (Figure 4B). In the cellular component category, endoplasmic reticulum lumen and lysosomal lumen were the most predominant functional terms encompassing three key genes: *VCAN*, *SDC2*, and *ARSA* (Figure 4C). Additionally, *GDF15*, *LIF*, *IL6*, *INHBA*, *HBEGF*, *NTF3*, *AREG*, *EREG*, *PPBP*, and *BMP2* were enriched by molecular functions that were mainly related to cytokine activity, chemokine activity, and growth-factor activity (Figure 4D). A KEGG-pathway-enrichment analysis was performed to further reveal how the cryopreservation caused biological-function and metabolic-activity changes. The DEGs were assigned to 50 KEGG pathways (Table S4) and a number of important pathways related to environmental information processing, organismal systems, cellular processes, and metabolism are exhibited in Figure 5. These include cytokine–cytokine-receptor interaction, the MAPK signaling pathway, the PI3K-Akt signaling pathway, the hippo signaling pathway, the NOD-like receptor signaling pathway, the lysosome, and the TNF signaling pathway.

### 3.5. Validation of RNA-Sequencing Data

**Figure 6:**
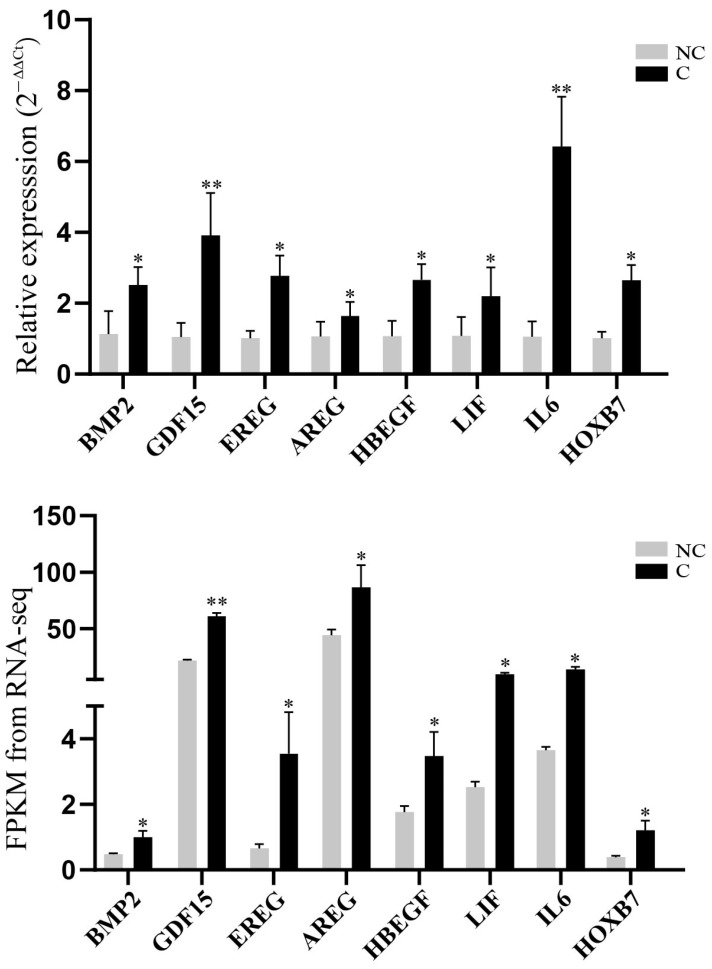
The expression patterns of DEGs detected by qRT-PCR and RNA-sequencing analysis. “*” and “**” represent adjusted *p* < 0.05 and adjusted *p* < 0.01, respectively. The error bars were generated based on standard deviation.

To verify the gene-expression levels quantified by RNA sequencing, eight DEGs, *BMP2*, *HOXB7*, *HBEGF*, *AREG*, *EREG*, *LIF*, *IL6*, and *GDF15*, highlighted by functional enrichment analysis were selected for qRT-PCR detection. The results showed that the differential expression trends of the selected genes in the cryopreserved and non-cryopreserved fibroblasts were in line with the differential expression patterns determined by the RNA sequencing (Figure 6), which indicated that our sequencing data were reliable.

## 4. Discussion

We report herein the isolation and culture of fibroblasts from porcine ear skin. After cryopreservation, the cryopreserved fibroblasts exhibited good post-thaw survival, and a non-significant alteration in cell viability was observed at the end of the 7 days of in vitro culture. In addition, 867 differentially expressed genes (DGEs) and related cell-recovery and -survival molecular processes were identified. Cell-type confirmation is an essential process before cryopreservation. In the present study, the cultures obtained from the ear skin grew with a typical morphology of fibroblasts and expressed intermediate filament protein vimentin (Figure S1 and Figure 1), which confirmed that ear skin can be regarded as a common source of fibroblast separation [23]. The post-thawing cellular-viability measurement is a key step in the evaluation of the success of cryopreservation. Our results showed that the post-thaw survival rate of cryopreserved cells was over 90%, and these cells exhibited very similar growth profiles to those of the non-cryopreserved cells, which implied that the proliferation capacities of the fibroblasts were maintained after thawing (Figure 2). Our findings were in accordance with the observations obtained in previous studies [24,25], which suggested that cryopreservation should be an effective approach to conserving the biobank of fibroblasts.

It is widely acknowledged that cryopreservation can trigger alterations in the gene-expression profiles of cells [26]. In light of our RNA-sequencing results, there were 632 up-regulated genes and 235 down-regulated genes in the cryopreserved fibroblasts (Figure 3). The GO enrichment analysis revealed that the majority of the genes were related to cellular-development processes (e.g., tissue and tube development), cellular components (e.g., endoplasmic reticulum and lysosomal lumen), and signaling-molecule activities (e.g., cytokine, chemokine, and growth-factor activity) (Figure 4). Furthermore, several specific KEGG pathways, such as cytokine–cytokine-receptor interaction, the MAPK signaling pathway, the PI3K-Akt signaling pathway, the hippo signaling pathway, and the NOD-like receptor signaling pathway, were enriched by most of the DGEs (Figure 5). Importantly, some of the key DGEs enriched in multiple GO and KEGG functional processes were detected. 

The bone morphogenetic protein 2 (*BMP2*) gene is a member of the transforming growth factor-β (TGF-β) superfamily. Normally, it is able to regulate fibroblast proliferation and differentiation [27,28]. A more recent study reported that the overexpression of *BMP2* can inhibit reactive oxygen species (ROS) generation to protect osteoblasts from oxidative stress [29]. This can be used to explain why the enhanced expression of BMP2 may exert a similar protective role in cryopreserved fibroblasts against cryopreservation-induced oxidative damage. The growth differentiation factor 15 (*GDF15*) gene is another member of the transforming growth factor-beta (TGF-β) superfamily; it is highly expressed in response to conditions associated with mitochondrial stress [30]. Additionally, cryopreserved somatic cells often suffer mitochondrial injury [31,32]. Hence, the abundant *GDF15* may be implicated in saving cryopreserved fibroblasts from mitochondrial damage. 

The epiregulin (*EREG*), amphiregulin (*AREG*), and heparin-binding EGF-like growth factor (*HBEGF*) genes are epidermal growth factors (EGFs) secreted by fibroblasts and act as important mitogenic mediators by binding to EGF receptors [33,34]. Intriguingly, evidence of these EGFs dealing with mechanical-stress stimulus was also proposed in previous studies. For instance, elevated expressions of *EREG*, *AREG*, and *HBEGF* were observed in bronchial epithelial cells after mechanical stimulus, and this response was amplified via the MAPK signaling pathway [35,36]. In pulmonary alveolar epithelial and endothelial cells, mechanical-stress exposure led to the augmented expression of *AREG*, which further activated the PI3K-Akt pathway [37]. Similarly, greater EGFs expression levels and activities of related pathways were exhibited in the cryopreserved fibroblasts, which could be linked to the mechanical stress triggered by cryopreservation.

Both leukemia inhibitory factor (*LIF*) and interleukin 6 (*IL-6*) are pleiotropic cytokines belonging to the *IL-6* superfamily [38]. Although the pro-inflammatory roles of *LIF* and *IL-6* were largely investigated in pathological conditions [39], their positive effects on cell viability have also been. Stoecklein et al. indicted that *LIF* had the potential to alleviate the cryo-injuries to embryo cells by modulating lipid metabolism, improving cytoskeleton integrity, and decreasing DNA fragmentation [40]. Jin et al. demonstrated that *IL-6* improved the recovery and survival of hepatocytes by enhancing proliferation pathways, reducing oxidative stress, and maintaining mitochondrial function [41]. Thus, the augmented expression of *LIF* and IL-6 might contribute to the non-significant alterations in cellular viability of the cryopreserved fibroblasts in comparison with the control fibroblasts after the one-week culture in our study.

The homeo box 7 (*HOX7*) gene belongs to the *HOX* family of transcription factors and the regulatory roles in proliferation, differentiation, and senescence of mesenchymal stem cells (MSCs) have been investigated [42,43]. In general, MSCs show a fibroblast-like morphology, while fibroblasts are defined as adherent cells of mesenchymal origin that share the mesenchymal phenotypes with MSCs [44]. Due to these striking similarities, we speculated that *HOX7* may also be associated with the cell fates of fibroblasts. Additionally, the declined expression of *HOX*-gene-family members was linked to the increased metabolic and oxidative stress of fibroblasts in the pathological state [45], which implied that the reinforcement of the expression of *HOX7* in cryopreserved fibroblasts might alleviate the cellular stresses triggered by cryopreservation. 

Based on our findings, various cellular stresses induced by the cryopreservation process should be the main constraints when cryopreserving porcine fibroblasts. Hence, several efforts could be made to improve the success rates of porcine-fibroblast cryopreservation. Firstly, the addition of appropriate cryoprotective agents is regarded as a necessary step to protect cells from mechanical and osmotic stress. The second action that must be taken during the cryopreservation process is setting the optimal cooling and thawing rates to prevent the formation of intracellular ice [4,46]. In addition, cryopreservation media should be supplemented with different substances, such as enzymatic and non-enzymatic antioxidants, to reduce the adverse effects of oxidative damage on cell viability and functionality.

There are some limitations in the present study. On one hand, our attention was fixed on the alterations in the gene-expression profiles of post-thaw fibroblasts, but the information related to the changes in the cooling and freezing stages were lacking. However, a comprehensive understanding of the main changes in the gene-expression patterns at each stage of the cryopreservation process can assist in the improvement of these procedures. On the other hand, although the identified key DEGs were intimately associated with the cell responses to various stresses reported in previous research, observation data for specific stresses were not obtained in the cryopreserved fibroblasts, which resulted in the failure to establish the relationships between the DEGs and the stresses caused by cryopreservation.

## 5. Conclusions

In conclusion, our results suggested that porcine ear skin should be regarded as a source of morphologically stable fibroblast isolation. The cryopreserved fibroblasts exhibited good post-thaw survival. Moreover, the cryopreservation did not significantly affect the proliferation capacities of the fibroblasts after 7 days of in vitro culture. This may have been due to the alterations in the gene-expression profiles and molecular functions related to the recovery and survival of the fibroblasts. Importantly, several key DEGs, namely *BMP2*, *GDF15*, *EREG*, *AREG*, *HBEGF*, *LIF*, *IL-6*, and *HOX-7* could potentially be utilized to improve the efficiency of fibroblast cryopreservation, although the underlying mechanisms responsible for their modulatory roles are not fully understood at present.

## Figures and Tables

**Table 1:** Sequence quality and read mapping of different groups.

Groups	Cryopreserved	Non-Cryopreserved
Total clean reads	151,935,710	142,796,298
Q30%	94.56%	94.30%
GC%	52.26%	52.26%
Mapped reads	145,743,081 (95.92%)	136,954,840 (95.90%)
Unique mapped reads	140,741,266 (92.63%)	132,860,276 (93.04%)

## Data Availability

The raw data supporting the conclusions of this article are available at China National GeneBank DataBase (https://db.cngb.org/, accessed on 14 November 2022) with accession number: CNP0003719.

## Supplementary Materials

The following supporting information can be downloaded at: https://www.mdpi.com/article/10.3390/genes14030751/s1. Figure S1: Morphological observation of cells under light microscope. Table S1: Primers used for qRT-PCR validation of selected DEGs. Table S2: The differential expressed genes (DEGs) between cryopreserved and non-cryopreserved cells. Table S3: GO enrichment analysis of DEGs. Table S4: KEGG-pathway-enrichment analysis of DEGs.
