# Supplementary figures and images for: Evaluating the Effects of Cryopreservation on the Viability and Gene Expression of Porcine-Ear-Skin Fibroblasts
Source: Genes (Basel). 2023 Mar 20;14(3):751. doi: 10.3390/genes14030751 (PMC10048577; doi:10.3390/genes14030751)

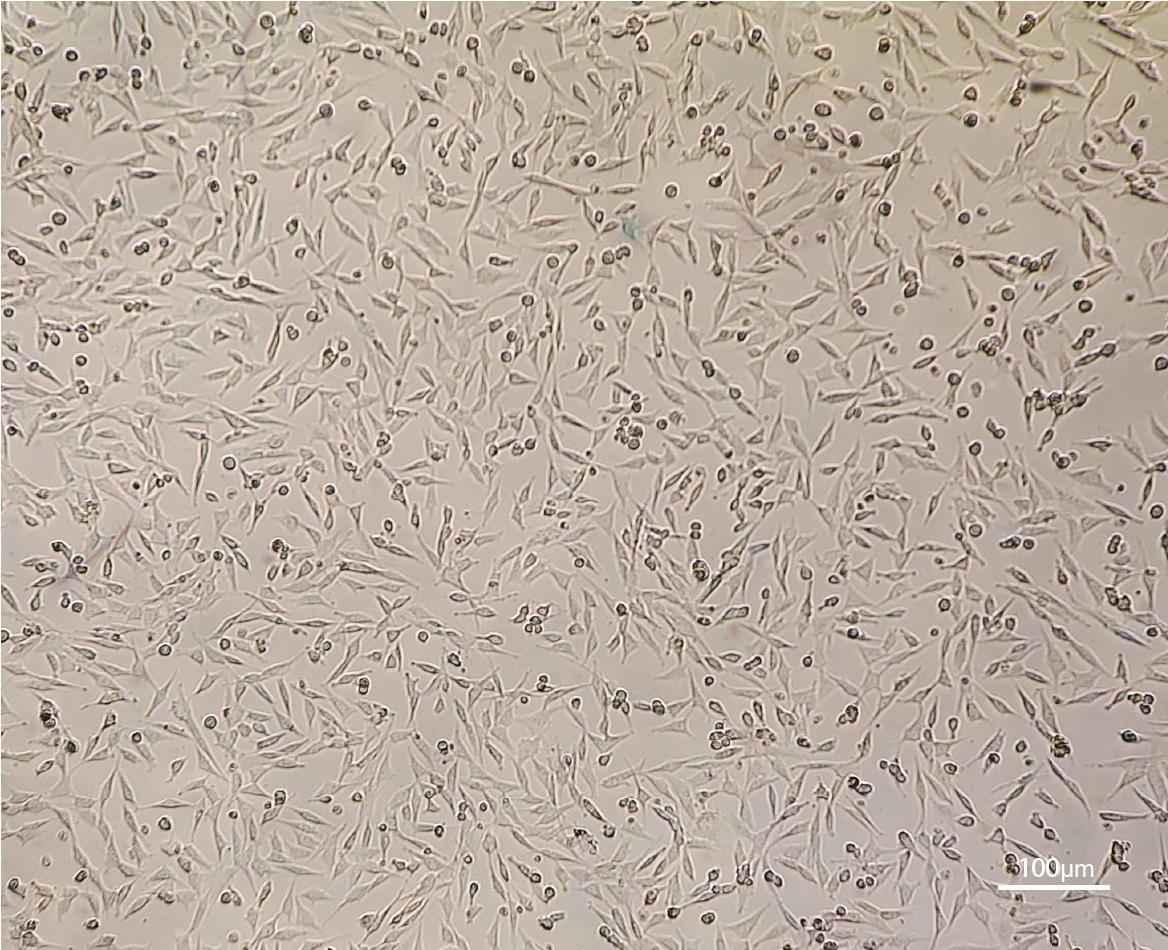

Supplement: Supplementary file 1 [file genes-14-00751-s001.zip › supplementary materials/Figure S1.tiff]
